# Multimodal Approach for Enhancing Biometric Authentication

**DOI:** 10.3390/jimaging9090168

**Published:** 2023-08-22

**Authors:** Nassim Ammour, Yakoub Bazi, Naif Alajlan

**Affiliations:** Computer Engineering Department, College of Computer and Information Sciences, King Saud University, Riyadh 11543, Saudi Arabia; nammour@ksu.edu.sa (N.A.); najlan@ksu.edu.sa (N.A.)

**Keywords:** fingerprint, multimodal fusion, presentation attack detection, heartbeat signal

## Abstract

Unimodal biometric systems rely on a single source or unique individual biological trait for measurement and examination. Fingerprint-based biometric systems are the most common, but they are vulnerable to presentation attacks or spoofing when a fake fingerprint is presented to the sensor. To address this issue, we propose an enhanced biometric system based on a multimodal approach using two types of biological traits. We propose to combine fingerprint and Electrocardiogram (ECG) signals to mitigate spoofing attacks. Specifically, we design a multimodal deep learning architecture that accepts fingerprints and ECG as inputs and fuses the feature vectors using stacking and channel-wise approaches. The feature extraction backbone of the architecture is based on data-efficient transformers. The experimental results demonstrate the promising capabilities of the proposed approach in enhancing the robustness of the system to presentation attacks.

## 1. Introduction

Biometrics, or human measurements, are metrics related to unique person characteristics that distinguish one individual from another. These biological characteristics can be used as biometric identifiers to recognize and verify a person’s identity. Physiological identifiers include fingerprints, face, palm, iris, and hand geometry, while behavioral identifiers include characteristics such as gait, hand-written signature, and voice. Biometrics technology involves measuring and processing these distinctive biological traits of individuals. Biometric authentication, on the other hand, is a security process that relies on these unique features to verify a person’s identity and ensure that they are who they claim to be.

The selection of a specific biometric identifier for a particular application depends on various factors such as uniqueness, permanence, universality, measurability, and performance [1]. Fingerprints are a reliable solution that are widely used due to their convenience, collectability, and high level of security [2,3]. However, the widespread use of fingerprint-based biometric systems has led to an increase in presentation attacks (PAs), where an artificial copy of a fingerprint is presented to the sensor to bypass the system’s security measures. This type of attack is known as a Presentation Attack Instrument (PAI) and is defined by ISO/IEC 30107 [4]. PAIs can be made from a range of materials and can be either artificial or synthetic fingerprint samples. To address the security concerns caused by these attacks, several automated presentation attack detection (PAD) techniques have been developed in recent years [5,6,7,8,9,10]. PAD systems are used to determine whether a specimen is from a genuine subject or an artificial copy (PA or artifact). In general, a combination of hardware and software technologies are used in PAD systems to detect a non-genuine or spoof presented biometric [11]. The reliability of the sensor used to measure the biometric trait and the degrees of freedom of the extracted features greatly affect the performance of the biometric system.

Recently, the Electrocardiogram (ECG) signal of the heart has been proposed as a biometric trait. It has been coined the “heartprint” [12], and similar to the fingerprint, it is a unique human trait and can be used to identify and authenticate subjects [13,14]. Compared to many other biometric modalities, the heartprint is shown to be the most promising [15]. The main advantage of the heartprint biometric is the fact that it verifies the liveness of the subject, making it more robust to spoofing attacks [16,17]. Another advantage is that the heartprint is a one-dimensional data signal, making it computationally more efficient than video or image-based biometric systems [18]. Considering that the heart electrical activity signal is a unique physiological signal existing only in a live person, it can be used to counter forge and fraud attacks. We will use the heartprint as a second modality to reinforce the detection of the artifact fingerprint sample.

These features make it suitable to combine heartprints with traditional biometric modalities like fingerprints, which is the main motivation for the method in this paper. Compared to unimodal biometric systems, multimodal systems are a combination of two or more biometric traits for improved recognition rate and protection against spoof attacks [19,20,21,22]. Fingerprints and heartprints could be a natural combination for multimodal fusion as high-quality heartprints can be captured from the fingers [23,24,25] simultaneously with fingerprints. In fact, the liveness property of heartprints makes them a secured biometric modality, and its fusion with fingerprints may yield a robust and secure authentication and identification system [26,27,28,29]. Several multimodal biometric systems which fuse fingerprints and heartprints have been introduced in the literature. Pouryayevali et al. [11] developed a sequential score fusion algorithm to fuse these to modalities. In this method, first, a heartprint matcher was used to authenticate a subject, and if accepted, his/her fingerprint was authenticated. Then, fusion was performed by employing a user-weighting score fusion method. Komeli et al. [28] proposed a multimodal system by fusing these two modalities with automatic template updating of heartprint records. Jomma et al. [30,31] used a sequential method to boost the robustness of fingerprint authentication against presentation attacks via fusion with a heartprint.

Recently, a limited number of attempts have been made to use deep learning for the fusion of these two modalities. Hammad et al. [27,32] fused heartprints and fingerprints using a convolutional neural network (CNN) based on different levels of fusion such as feature-level fusion and decision-level fusion. CNN was used for feature extraction from the individual modalities, and they were combined using internal fusion to generate the biometric templates which were used by a classifier for authentication. Jomaa et al. [33] presented a multimodal biometric method for presentation attack detection. Their method is based on an end-to-end deep learning model that accepts fingerprint and heartprint modalities at the same time. EfficientNets were used for generating a fingerprint feature representation. On the other hand, a 2D convolutional neural network (2D-CNN) was used to convert the heartprint into a 2D image which was fed to Mobilenet-v2 layers for feature presentation. The concatenated features from both network branches are then fed into an additional fully connected layer followed by a Swish activation function and a dropout regularization layer. Finally, a binary classifier was used to classify the multimodal sample into artefact or bona-fide classes.

In this paper, we propose a multimodal biometric solution which combines fingerprint with heartprint modalities. Due to the lack of a multimodal dataset that combines fingerprints and heartprints, we made our own by combining a mini-LivDet2015 dataset and the newly released Heartprint dataset [12]. We used fingerprint images sensed using the digital Persona sensor, which is the most difficult subset of the LivDet2015 dataset, and we call it the mini-LivDet2015 dataset. It comprises 70 subjects or individuals, and each individual has 10 bona-fide and 12 artefact fingerprint images. To construct the multimodal dataset, we randomly associated each person from the mini-livdet2015 dataset to a person from the Heartprint dataset. Then, we designed a deep learning architecture that is composed of two branches: one that accepts a fingerprint input, while the other accepts a heartbeat input. As in previous work, we first convert the heartprint 1D input into an image using a special CNN model. Then, unlike previous work in the literature, we use a Data Efficient Image Transformer (DEIT) model as the backbone for feature extraction. In addition, we fuse the feature vectors extracted for each input using a channel-wise fusion approach and compare it to the typical stacking approach.

The feature vectors extracted by each branch are fused using two approaches: stacking or channel-wise fusion.

Our contributions via this paper can be summarized in the following points:

- We propose an end-to-end deep learning model for a multimodal biometric method using image transformers.
- The proposed method employs a channel-wise fusion approach which improves performance compared to the typical stacking approach.

The remainder of the paper is organized as follows. Section 2 provides materials and methods. Then, Section 3 presents the results that show the capabilities of our method on common RS scene datasets. Finally, we give conclusions and future research directions in Section 4.

## 2. Materials and Methods

The purpose of typical fingerprint PAD is to identify whether a fingerprint image is an artefact (PA) or a bona-fide fingerprint sample. Given the fingerprint dataset, $D={\left\{X_{i},y_{i}\right\}}_{i=1}^{N=A+B}$ containing A artefact samples and B bona-fide samples, where $X_{i}$ represents the fingerprint input image and $y_{i}$ is a binary label denoting if a fingerprint is an artefact or a bona-fide. In this research work, we add a heartprint signal as an extra input modality. Thus, the obtained dataset is a triplet data $D={\left\{\left(X_{i}^{f},X_{i}^{e}\right),y_{i}\right\}}_{i=1}^{N}$, where $X_{i}^{e}$ is the heartprint signal and $X_{i}^{f}$ is the fingerprint image.

**Figure 1:**
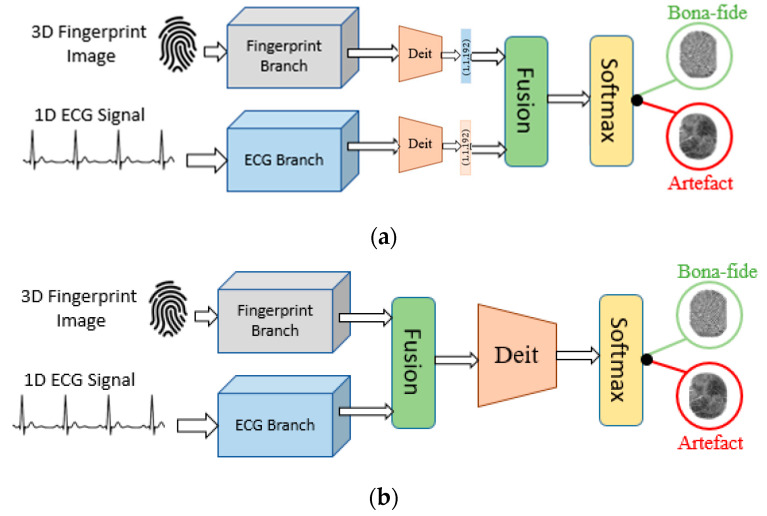
Overall architecture of the proposed deep learning model for multimodal biometric analysis: (**a**) feature concatenation fusion approach; (**b**) channel-wise fusion approach.

As shown in Figure 1, the proposed multimodal deep learning model includes four main parts: fingerprint and heartprint preprocessing and conditioning, fusion, feature extraction, and the task layer. The first module is a data preprocessing block used to perform data conditioning on each modality using different techniques, as each modality possesses distinct characteristics. Then, a fusion process is adopted to combine the data from each modality. As the feature extraction backbone, we used an attention-based deep learning DEIT model to extract discriminant features from the two modalities. Lastly, a classification layer identifies the output class. 

### 2.1. Fingerprint Block

Fingerprints, part of the dermatoglyphics field, are a complex and unique pattern of curving line structures called friction ridges. The number, shape (loops, whorls, and arches), and location of each ridge make every person unique, and they do not vary with growth or age. The fingerprint image consists of dark lines called ridges and white lines called valleys. In this work, we consider the International Fingerprint Liveness Detection Competition 2015 (LivDet 2015) dataset.

### 2.2. Heartprint Block

**Figure 2:**
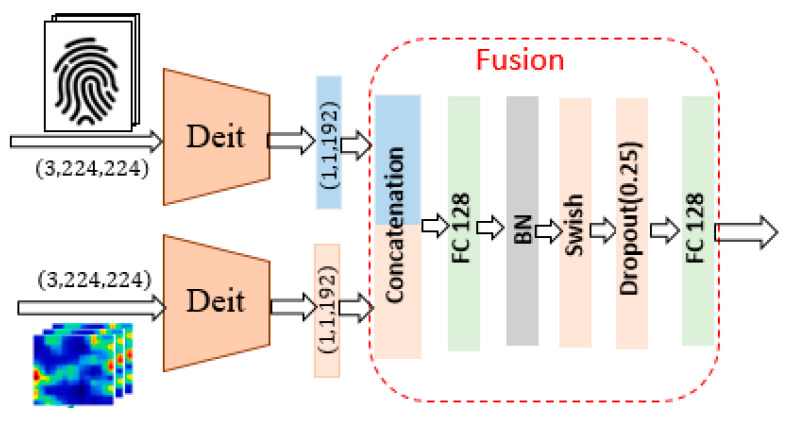
Illustration of the fusion approach based on feature concatenation.

The Heartprint2022 database contains a different number of heartbeat samples with a length of 740 each for 199 persons. We propose to extract features from the heartprint and convert it to an image, which is needed for the fusion process. As shown in Figure 2, a signal process of three sub-blocks produces a feature image of the size $\left(3,224,224\right)$ as an output of the heartprint branch. The first sub-block concatenates five heartbeats chosen randomly from the selected person. The second sub-block generates a 2D time-frequency analysis image from the 1D heartprint input signal by applying the short-time Fourier transform (STFT). STFT is a sequence of Fourier transforms applied on a signal using a window for segmented analysis. For situations in which frequency components change over time, the STFT provides time-localized frequency information. The STFT is given by the Fourier transform of the windowed signal $x\left[n\right]w\left[n-m\right]$, where $x\left[n\right]$ denotes the signal and $w\left[n\right]$ denotes an N-point window function.

(1) $$X\left[m,k\right]=\sum_{n=0}^{N-1}x\left[n\right]w\left[n-m\right]e^{-j2\pi nk/N}$$

The third sub-block in the heartprint branch is 2D CNN architecture converts the STFT input image of the size $\left(26,37\right)$ to a $\left(3,224,224\right)$ 3D feature image. 

### 2.3. Feature-Concatenation-Based Fusion Module

The first fusion strategy is based on the concatenation of the extracted features of the fingerprint image and the heartprint signal. The architecture of the two-branch neural network is illustrated in Figure 2, which contains the fingerprint branch for the feature extraction and the heartprint branch for height-relevant feature learning. The feature fusion module consists of a sequence of deep learning layers. The first layer applies the concatenation of the feature vector, arriving from the fingerprint feature extraction module and the feature vector received from the heartprint feature extraction module. The combined feature passes through a fully connected layer followed by a batch normalization layer, a Swish activation layer, a dropout layer, and a second fully connected layer. Finally, a binary classifier decides in which category, artifact or bona-fide, the elaborated fingerprint-heartprint feature belongs to.

### 2.4. Channel-Wise Fusion Module

**Figure 3:**
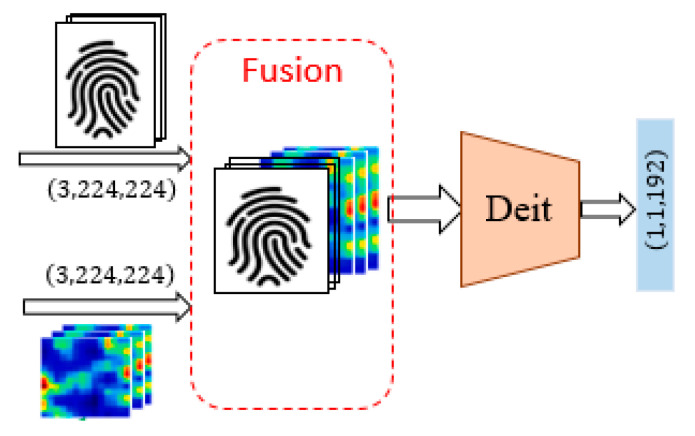
Structure of the channel-wise fusion module.

Multimodal biometric systems seek to increase performance that may not be possible by using a single biometric indicator by providing multiple shreds of evidence of the same identity. An optimal fusion of multiple modalities is a fundamental request for the development of a reliable solution. In attempting to improve the performance of the detection system, the outputs of the fingerprint and heartprint branches are further processed using a fusion module. This fusion module is performed by intercalating the heartprint SIFT image as additional bands to the fingerprint image. We call this a channel-wise fusion approach and illustrate it in Figure 3.

### 2.5. Feature Extraction Module

A wide variety of deep learning strategies have been used to build biometric identification systems. Usually, these methods depend on CNN to extract features from input data. Inspired by the biological systems of humans, the attention mechanism has revolutionized the natural language processing and computer vision systems [1,2]. The attention mechanism has reasonably become one of the most fundamental concepts in the deep learning field. The feature extraction module uses a state-of-the-art data-efficient ViT variant (Deit) to extract discriminative features for image classification tasks. Deit has the same architecture as ViT.

The input image to the ViT is split into N patches of a fixed size D, $X\in R^{N\times D}$, the patches are flattened and fed to a linear projection to create lower-dimensional linear embeddings, a positional embedding is added with the class of the embedded image, and the sequence is fed to the transformer encoder. The transformer encoder uses a Multi-Head Self Attention layer (MSA) as an attention mechanism in between all the input vectors. The input to the attention block has three linear input layers (receive the queries $Q=XW_{Q}$, keys $K=XW_{K}$, and values $V=XW_{V}$ with $W_{Q}$,$W_{K}$,$W_{V}\in R^{D\times d}$ are the parameters of the linear transformations), followed by a scaled dot-product attention function to give the output matrix:

(2) $$Attention\left(Q,K,V\right)=Softmax\left(QK^{T}/\sqrt{d}\right)V$$

where the term $\sqrt{d}$ provides proper normalization. The attention function is repeated h times to produce a multi-head self-attention (h heads), a concatenation operation joins the h outputs of the different heads, and a final MLP head performs the classification task. 

Introduced by Facebook AI, the structure of the Deit model, built based on the ViT model [34], showed enhancement over previous ViT models. ViT does not generalize well when trained on a small amount of data and needs to be pre-trained with a huge amount (hundreds of millions) of images. Deit architecture is proposed using a ViT architecture with a teacher-student strategy and a distillation token. The distillation token, which allows the deep model to learn from the teacher’s output, interacts with the class token and patch tokens through the self-attention layers to provide the hard label predicted by the teacher. 

## 3. Results

To evaluate the proposed method, we first built our own multimodal dataset. Then, we split the dataset into training and testing sets, by splitting based on the subjects.

### 3.1. Dataset Description 

The International Fingerprint Liveness Detection Competition 2015 (LivDet 2015) and the real heartprint dataset, called Heartprint2022, are used to evaluate and validate the performance of the proposed deep learning architecture. The LivDet 2015 dataset is provided by Orrù et al. and can be downloaded from [6], whereas the Heartprint2022 dataset is a new dataset collected in our lab, which is the Advanced Lab for Intelligent Systems Research (ALISR), and can be downloaded from here [12]. 

The LivDet 2015 dataset has approximately 19,000 fingerprint images captured using four different optical fingerprint sensors: GreenBit, Biometrika, Digital Persona, and CrossMatch [6]. It aims to develop both software-based and hardware-based fingerprint liveness detection methodologies [6]. LivDet 2015 contains a training set dataset and a testing dataset [7]. Each set contains bona-fide (live) and artefact (fake) fingerprint images acquired via different fingerprint scanners, as illustrated in Table 1. To mimic real scenarios, the image capturing process includes normal mode, with dry and wet fingers, and with high and low pressure.

**Figure 4:**
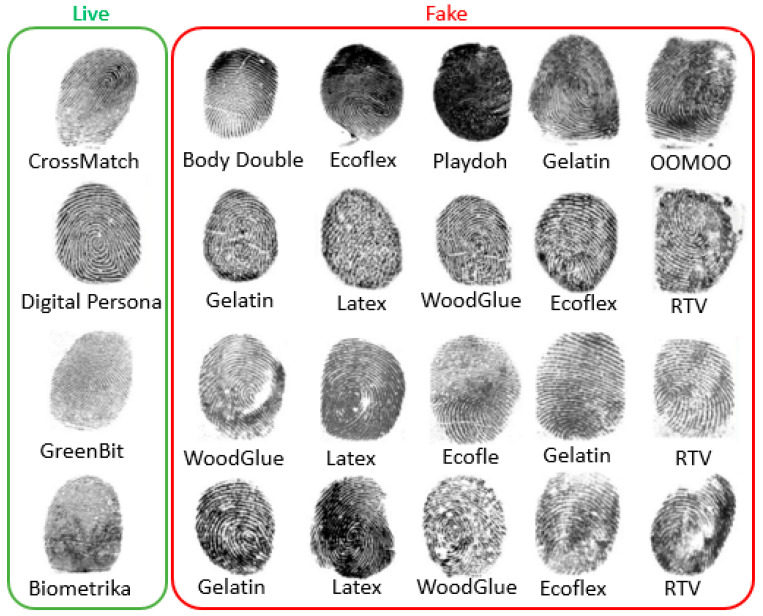
Sample images of LivDet2015 dataset captured using CrossMatch, Digital Persona, GreenBit, and Biometrica sensors. Live samples are in the green box and fake samples made of different materials are in the red box.

LivDet 2015 datasets contain spoof fingerprint images collected using artificial fingers. Artificial fingers are fabricated using plasticine-like material to create a negative impression or a mold of the real finger (cooperative method), the mold is then filled to produce the artificial finger using gelatin, PlayDoh, or silicone. A latent fingerprint left on a surface is another way to make artificial fingerprints (non-cooperative method); a transparency sheet is obtained from a processed latent fingerprint and used to create the mold. Figure 4 shows samples from the LivDet 2015 dataset.

LivDet 2015 dataset contains spoof images made using diverse materials, such as Ecoflex, gelatin, latex, wood glue, liquid Ecoflex, and RTV (a two-component silicone rubber), as shown in Table 2. The testing set includes some spoof images of materials which were not included in the training set.

**Figure 5:**
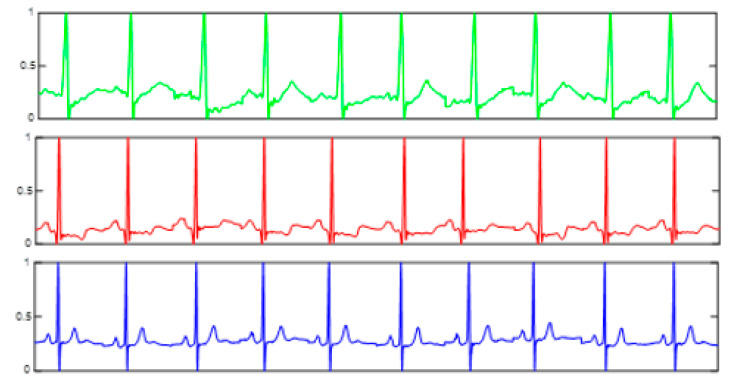
Heartprint sample of 10 heartbeats from three different subjects.

The heartprint dataset is collected using the ReadMyHeart ECG device, by DailyCare BioMedical [35]. The ReadMyHeart handheld ECG is simple to use without skin electrodes, leads, wires, or conductive gels. The measurements are taken by placing the thumbs on the conductive plates as shown in Figure 5. The heartprint needs only 30 s of measuring time during which 15 s are digitalized and exported to the computer via a USB port. The heartbeat activities of 164 persons are captured during two sessions to build an Heartprint2022 dataset of 656 ECG records. During the preprocessing step, the authors used a four-order band-pass Butterworth filter with cut-off frequencies of 0.25 and 40 Hz to remove the different types of noise that can affect the heartprint, such as the power-line interface, baseline wanders, and patient-electrode motion artifacts.

The non-existence of public multimodal datasets encompassing heartprint and fingerprint signals incited us to build a multimodal dataset from the LivDet 2015 dataset and Heartprint dataset. To this end, we first detect the heartbeats from each heartprint record in Heartprint using an effective curvature-based technique [36]. Then, to create the heartprint modality for our experiments, we randomly select a few heartbeats from the heartprint records of each subject. 

We used a subset of 70 subjects from the LivDet 2015 dataset, each of which has 10 bona-fide images from the Digital Persona sensor and 12 artefact images to construct a mini-LivDet 2015 dataset. Then, we associated a random subject from the Heartprint2022 dataset to each subject from the mini-LivDet 2015 dataset. If the fingerprint image is fake, the subject must have different heartprints. To substantiate this fact, we assign a random set of heartbeats from the same subject to the bona-fide images of one person, and we assign the random set of another randomly selected person to the artefact fingerprint of the same person. The new multimodal dataset containing fingerprint images and heartbeat signals of 70 subjects is described in Table 3.

During the training phase, the model receives data batches consisting of both classes, the bona-fide fingerprint samples with bona-fide heartbeat samples and fake fingerprint samples with bona-fide heartbeat samples of another person. In this way, the deep model learns to discriminate between the fake fingerprint sample and the bona-fide fingerprint sample.

### 3.2. Experimental Setup and Performance Metrics

We conducted different experiments to evaluate the performance of the proposed methodology and compare our results with previous state-of-the-art methods. In the first experiment, we trained the deep model using only the fingerprint branch to assess its performance regarding the detection of PAs. The model is trained using the training subset of the LivDet 2015 dataset. Then, we trained and evaluated the model on the multimodal dataset, using the proposed fusion strategies to detect and prevent PAs.

To check the reproducibility of the proposed deep learning model, all experiments are repeated several times and the average classification accuracy is reported. All experiments are implemented in Python and the PyTorch machine learning library using a workstation that has a Core i9 processor with a speed of 3.6 GHz, 64 GB of memory, and GPU with 11 GB GDDR5X memory. 

### 3.3. Results and Discussions

In the first experiment, we trained different deep models on the multimodal dataset. The average detection accuracy of the different models using a single modality (no fusion) and multimodality (with fusion) biometric traits is reported in Table 4. In this experiment, we achieved the fusion process between the contribution of the fingerprint and the heartprint information part via concatenation at the feature level. We employed different pre-trained models as the backbone networks to extract the feature of each modality. We trained the different models on the fingerprint images and heartprint signals using the Adam optimizer with a scalable learning rate, a batch size of 32, and 30 training epochs.

We note from the reported results in Table 4 that the Resnet50 architecture outperforms the other CNN models and achieves the highest accuracy of 98.7%. Resnet18 and mobilenetv2_110d architectures perform with a high accuracy of 98.3%, which is not far off the highest performance. Deit_tiny_patch16_224_fe and vit_tiny_patch16_224 architectures achieve the lowest accuracies of 95% and 97.1%, respectively. 

In the second experiment, we applied the fusion between the fingerprint and the heartprint signal at the data level. We combined the two modalities by inserting the heartprint image as a new channel in the fingerprint image. We trained different pre-trained models on the combined fingerprint-heartprint to extract significant features from the combined images.

As shown in Table 4, combining fingerprints with heartprint data provides better performance in detecting fake fingerprints than single modality biometric traits. The different deep models, namely, Deit_tiny, Resnet18, Resnet18d, Resnet50, MobileNetv2_100, and MobileNetv2_110d, vit_tiny_patch16_224, perform with accuracies of 98.8%, 99%, 97.6%, 98.3%, 98.3%, 98.6%, and 98.1%, respectively. We can clearly see that the multimodal biometric system outperforms a biometric system with a single biometric indicator (i.e., without using a fusion). This performance is achieved thanks to converting the heartprint to a 2D image and the benefit of the 2D convolution’s power in the deep learning models. The Resnet50 deep model achieves the highest performance (an accuracy of 99%), surpassing the other models.

A. *Sensitivity Analysis of the Number of Training Subjects*

Generally, it is common knowledge that a small training dataset produces weak approximation [37]. To assess the impact of the training set size on the system performance, we trained and evaluated the different models on a dataset with different sizes (between 20% and 80%) and reported the achieved performance of each model in Table 5.

The reported results in Table 5 reveal that increasing the size of the training dataset in the learning process improves the classification performance during the testing phase. We can observe this behavior from the models’ performances. Deit-tiny performs well when trained on a dataset with a size more than 50%. With a small size of training samples (20%), the Resnet18 and Resnet50 models achieve good accuracies and maintain their performances for all the training sample sizes. As shown in Table 5, Resnet18 reaches an accuracy of 99.3% and outperforms all the other deep learning models when trained on 80% of the dataset. It is well known that training a deep learning model involves large amounts of labeled training samples. Training a model with insufficient amounts of labeled data degrades the testing accuracy. Despite training with small amounts of training samples, the deep models perform well with good accuracies (95.05%, 96.5%, 97%, and 90.5%) for Deit_tiny, Resnet18, Resnet50, and Mobilenetv2_100, respectively, when trained using only 20% of the dataset.

B. *Sensitivity of the heartprint feature*

During this experiment, we assess the effect of the number of heartbeats used in the STFT heartprint image on the model performance accuracy. We repeated the experiment with STFT heartprint images built using a different number of heartbeats (heartprint of length ranged between 5 heartbeats and 20 heartbeats), the model performance is reported in Table 6.

Table 6 shows that increasing the number of heartbeats in the STFT transformation of the heartprint to an image does not significantly affect the accuracy. The highest accuracy (98.7%) is obtained when adopting five heartbeats of the heartprint signal to construct the STFT image. 

## 4. Conclusions

In this research, we have presented an end-to-end deep learning multimodal fusion technique for a fingerprint presentation attack detection system. We introduced a variety of pre-trained deep learning models as feature learning backbone networks. The selection of an appropriate modality and the choice of an optimal fusion strategy are crucial factors in the performance of a biometric system. To enhance the performance of the PAD system, the heartprint is added as an additional modality, and two fusion strategies, i.e., heartprint-fingerprint feature concatenation and heartprint-fingerprint channel insertion, are adopted and tested. The obtained experimental results show that the fingerprint-heartprint multimodal PAD system achieved high accuracy, confirming the effectiveness of the proposed approach. For future developments, we propose to investigate other fusion strategies based on feature selection, feature weighting, and decision fusion to increase the robustness of the system. 

## Figures and Tables

**Table 1:** Device and image characteristics of the LivDet 2015 dataset.

Sensor	Model	Image Size [pixel]	Training		Testing	
			Live	Fake	Live	Fake
**Green Bit**	**DactyScan26**	500 × 500	1000	1000	1000	1500
**Biometrika**	**HiScan-PRO**	1000 × 1000	1000	1000	1000	1500
**Digital Persona**	**U.are.U 5160**	252 × 324	1000	1000	1000	1500
**Crossmatch**	**L Scan Guardian**	640 × 480	1500	1500	1500	1448

**Table 2:** Materials used for fabricating spoof images in the LivDet 2015 dataset. The unknown materials that do not exist in the training part are in bold.

Sensor	Training	Testing
**Green Bit** **Biometrika** **Digital Persona**	Ecoflex, gelatin, latex, wood glue	Ecoflex, gelatin, latex, wood glue, Liquid Ecoflex, RTV
**Crossmatch**	Body Double, Ecoflex, PlayDoh	Body Double, Ecoflex, PlayDoh, OOMOO, gelatin

**Table 3:** Materials used for fabricating spoof images in the LivDet 2015 dataset. The unknown materials that do not exist in the training part are in bold.

	Fingerprint Images		Heartbeats
	Bona-Fide	Artefact	
**# samples per subject**	10	12	10
**Total number of samples**	700	840	700

**Table 4:** Average accuracy of the proposed fusion by concatenation architecture.

Biometric Modality	CNN Architecture	Average Accuracy %	
		Concatenation	Channel-Wise
**Fingerprint** **(No fusion)**	Deit_tiny_patch16_224_fe	97.4	97.4
	Resnet18	98.0	98.0
**Fusion by Concatenation** **Fingerprint + ECG**	Deit_tiny_patch16_224_fe	95.0	98.8
	Resnet18	98.3	**99**
	Resnet18d	97.6	97.6
	Resnet50	**98.6**	98.3
	mobilenetv2_100	97.2	98.3
	mobilenetv2_110d	98.3	98.6
	vit_tiny_patch16_224	97.1	98.1%

**Table 5:** Average accuracy in terms of percentage of subjects used in the training set. Channel-wise concatenation approach is used.

CNN Architecture (Channel Fusion)	Percentage of Subjects Used for Training				
	20%	30%	50%	70%	80%
**Deit_tiny_patch16_224_fe**	95.05	95.36	97.4	97.84	98.8
**Resnet18**	96.5	98	97.4	98.4	99.3
**Resnet50**	97	96.6	98.8	99.1	97.2
**Mobilenetv2_100**	90.5	93.5	95.6	96.4	96.5

**Table 6:** Average accuracy of the proposed fusion via concatenation architecture with respect to the number of heartbeats.

Number of Heartbeats	Accuracy
5	98.70
7	98.05
10	96.83
13	97.40
15	98.05
18	98.38
20	96.75

## Data Availability

Not applicable.
